# Postural orthostatic tachycardia syndrome after surgical correction of an aortic coarctation: a case report

**DOI:** 10.1186/1752-1947-6-241

**Published:** 2012-08-13

**Authors:** Lucie Fernex, Alessandra Coeytaux, Thierry Rochat, Saziye Karaca, Stephen Perrig, Haran Burri, Mathieu R Nendaz

**Affiliations:** 1 Internal Medicine, Department of Internal Medicine, University Hospital of Geneva, Gabrielle-Perret-Gentil 4, Geneva, 11 1211, Switzerland; 2 Neurology, Department of Clinical Neurosciences, University Hospital of Geneva, Gabrielle-Perret-Gentil 4, Geneva, 11 1211, Switzerland; 3 Pneumology, Department of Internal Medicine, University Hospital of Geneva, Gabrielle-Perret-Gentil 4, Geneva, 11 1211, Switzerland; 4 Cardiovascular Surgery, Department of Surgery, University Hospital of Geneva, Gabrielle-Perret-Gentil 4, Geneva, 11 1211, Switzerland; 5 Neuropsychiatry, Department of Mental Health and Psychiatry, University Hospital of Geneva, Petit Bel-Air 2, Chêne-Bourg, 1225, Switzerland; 6 Cardiology, Department of Internal Medicine, University Hospital of Geneva, Gabrielle-Perret-Gentil 4, Geneva, 11 1211, Switzerland

## Abstract

**Introduction:**

We report a case of postural tachycardia syndrome occurring after the surgical correction of an aortic coarctation, and coexisting with upper airway resistance syndrome.

**Case presentation:**

A 29-year-old Caucasian man complained of extreme fatigue, daytime sleepiness, shortness of breath on exertion, light-headedness and general weakness on standing. These symptoms began shortly after the surgical correction of an aortic coarctation and became progressively more debilitating, impairing any daily activity. An extensive work-up revealed postural tachycardia syndrome and a coexisting sleep-related breathing disorder, characterized as upper airway resistance syndrome.

**Conclusion:**

This is the first reported case describing the occurrence of postural tachycardia syndrome after the surgical correction of an aortic coarctation. This case also provides evidence for the suggestion that this syndrome may coexist with upper airway resistance syndrome, although the exact nature of their relationship must still be better established.

## Introduction

Postural tachycardia syndrome (POTS) is a chronic form of orthostatic intolerance. It is characterized by an exaggerated increase in heart rate on standing, without any substantial drop in blood pressure [1]. In this report, we describe a young male patient who was admitted for POTS shortly after the surgical correction of an aortic coarctation. To the best of our knowledge, this is the first reported case of POTS arising after this type of surgery. Additionally, a coexisting sleep-related breathing disorder characterized as upper airway resistance syndrome (UARS) was diagnosed by an extensive sleep study. Diminished sleep quality in POTS has been reported in the literature [2] but has never been characterized by detailed polysomnography.

## Case presentation

Our patient is a previously asymptomatic 29-year-old Caucasian man who was diagnosed with isthmic aortic coarctation at the age of 28 years. At that time, he presented with progressive shortness of breath, significant chest pain and diminished exercise tolerance. His blood pressure was 150/90mmHg without treatment. Echocardiography showed dilatation of his ascending aorta (39×40mm) and a bicuspid aortic valve with mild insufficiency.

Our patient underwent surgery three months after his symptoms began. The surgical technique was resection of the long segment (4cm) of the coarcted aortic arch and a termino-terminal anastomosis of an intervascular number 16 polytetrafluoroethylene (PTFE) tube. Two thoracic collaterals were ligatured during the resection of the aortic segment. Surgery was performed without any immediate complication. The gradient of the coarctation was 60mmHg before surgery, and was normal after the procedure. After ten days of hospital stay, our patient was discharged. He was asymptomatic and his blood pressure under treatment with nifedipine (2×30mg/day) was 120/85mmHg.

During the eight months after his surgery, our patient presented the following new symptoms, which worsened very progressively until they impaired his normal daily functioning: shortness of breath on exertion, light-headedness and dizziness on standing, fatigue, excessive daytime sleepiness and hypersomnia (up to 16 hours a day). Sleep was not restorative and our patient described daytime somnolence. He could no longer attend his work. He also complained of abdominal cramps after meals and constipation.

An echocardiogram and thoracic computed tomography showed no relevant findings. Our patient developed depressive symptoms, which showed partial improvement after the introduction of venlafaxine (150mg/day). Other medications included amlodipine (5mg/day) and valsartan (80mg/day) for residual hypertension, and aspirin (100mg/day).

Eighteen months after his aortic surgery, our patient was finally admitted to our hospital because the same symptoms persisted and had worsened. A complete history revealed that he was not known for snoring, but his girlfriend reported leg jerks during sleep and sleep talking. He denied chronic nasal congestion or substance abuse. He drank two litres of cola-drink per day.

On physical examination, our patient could not stand for more than 10 minutes because of dizziness and a sense of weakness. His body mass index was 21kg/m^2^ and a small oral cavity with micrognathia was observed. He scored 19 out of 24 on the Epworth Sleepiness Scale, 6.6 out of 7 on the Fatigue Severity Scale and 14 out of 63 on the Beck Depression Inventory. Laboratory values were in the normal range, including thyroid-stimulating hormone and arterial blood gases. Adrenal insufficiency was excluded. Pulmonary function tests and cerebral magnetic resonance imaging were not contributory.

A polysomnography and a multiple sleep latency test detected breathing abnormalities, as detailed in Table 1. There were no periodic leg movements (index of periodic limb movements in sleep: 7) and narcolepsy was ruled out by the multiple sleep latency test (mean latency: 11min 33s). Thus, it was concluded that our patient had UARS together with micrognathia and excessive daytime sleepiness [3]. The UARS was severe enough to explain part of his symptoms, such as his extreme fatigue and functional impairment, but could not explain the orthostatic symptoms [4].

**Table 1:** Polysomnography


Central apnea (N)	1	Mean oxygen saturation (%)	95
Obstructive hypopnea (N)	104	Minimum oxygen saturation (%)	90
Respiratory effort-related arousals (N)	131	Morning pH	7.4
Apnea-hypopnea index	12.3	Morning partial pressure of CO_2_ (kPa)	6.5
Respiratory disturbance index	27.7	Morning partial pressure of O_2_ (kPa)	11
Mean heart rate (beats/min)	70	Morning HCO3^-^ (kPa)	30

An orthostatic measurement of his heart rate and blood pressure showed an important heart rate increment on standing (from 77 beats/min supine to 106 beats/min when getting up and 146 beats/min after 5min of standing). During the test, his blood pressure remained stable at a value of 115/83mmHg and our patient developed his usual symptoms of dizziness, shortness of breath, paresthesia and general weakness. After seven minutes, he could no longer tolerate the standing position. A head-up tilt test reproduced the same orthostatic symptoms, with an increased sinus rate and stable blood pressure (Table 2). The increase in heart rate without orthostatic hypotension along with the accompanying symptoms confirmed the presence of POTS. A 24 hour Holter monitoring excluded any significant arrhythmia.

**Table 2:** Head-up tilt test

**Time (minute)**	**Systolic blood pressure (mmHg)**	**Diastolic blood pressure (mmHg)**	**Mean blood pressure (mmHg)**	**Heart rate (beats/min)**
0 (supine)	118	85	94	69
2	118	94	99	87
4	124	93	102	99
6	119	83	97	93
8	121	82	94	93
10	123	80	94	99

Amlodipine and valsartan were stopped and propranolol was introduced (3×10mg/day), with some improvement of the orthostatic symptoms. Our patient entered an exercise rehabilitation program. He was encouraged to maintain a high liquid and salt intake.

Two months later, our patient was able to perform 70% of his normal activities of daily living under this treatment. He described nearly complete disappearance of hypersomnia, excessive daytime sleepiness and fatigue. Despite this initial improvement, our patient experienced a recurrence of the same symptoms after the following six months, albeit of reduced severity.

## Discussion

POTS typically affects patients aged 15 to 50 years [5]. The typical symptoms are posture-related and are relieved by lying down or sitting. They include light-headedness, weakness, dizziness, breathing difficulties, tremulousness*,* blurred vision, palpitations, anxiety and, occasionally, syncope. Our patient presented many of these symptoms. Exacerbation by exertion is also a common clinical feature. Non-orthostatic symptoms include fatigue, migraine and gastrointestinal tract symptoms (nausea, bloating, diarrhea, constipation, abdominal pain). The diagnostic criteria include the presence of orthostatic symptoms associated with a heart rate increase of 30 beats/min or greater within five minutes of standing or head-up tilt, without orthostatic hypotension. Typically, the heart rate increases to 120 beats/min [1].

The etiology of POTS is probably heterogeneous and the pathophysiology remains unclear. Three main groups have been identified: neuropathic, hyperadrenergic and POTS related to deconditioning [1]. In the neuropathic form, a restricted autonomic neuropathy with partial sympathetic denervation is present and predominates in the lower limbs [6], with an inability of the peripheral vasculature to constrict in response to orthostatic stress. The hyperadrenergic form of POTS is characterized by increased sympathetic activity of unknown origin, with orthostatic plasma norepinephrine values above 600pg/mL and a higher blood pressure on standing [7]. POTS related to deconditioning affects patients with a severe illness or any event impeding physical activity. It is due to excessive venous pooling in the legs and hypovolemia while standing [1]. Infection, surgery and traumas are frequent precipitating events.

Secondary forms of POTS occur in association with other disorders, most commonly diabetes mellitus. Other conditions include amyloidosis, sarcoidosis, alcoholism, systemic lupus erythematosus, Sjögren’s syndrome, heavy-metal intoxication, joint hypermobility syndrome, pure autonomic failure, multiple-system atrophy and paraneoplastic syndrome [7]. Therapy and prognosis are determined by the underlying causative disorder. Recently, Kanjwal *et al*. published a series of nine patients diagnosed with multiple sclerosis and POTS [8]. POTS after a traumatic brain injury, Lyme disease, atrioventricular nodal reentrant tachycardia ablation and electrical injury has also been described [9-12].

Our patient presented several risk factors for POTS. First, prolonged bed rest following coarctation repair may have been an initiating event for his physical deconditioning. Second, the antihypertensive drugs (amlodipine and valsartan) prescribed after surgery for residual hypertension, known to worsen orthostatic intolerance, may also have contributed to his condition. This could partly explain the improvement observed after discontinuation of these pharmacologic agents.

Our patient developed symptoms of POTS after the surgical correction of aortic coarctation. Beekman *et al*. described in 1983 an altered baroreceptor function with diminished baroreceptor sensitivity in a group of children with repaired aortic coarctation [13]. Functional changes of the aortic arch and carotid baroreceptors seem to be related to structural modifications of the precoarctation aorta, the wall of which contains more collagen and fewer muscular fibers, resulting in decreased compliance. In a more recent paper, other authors studied 45 patients with aortic coarctation repair and found frequent abnormal hemodynamic responses to orthostatic tests. Nine patients had a blood pressure decrease of more than 20mmHg, while two patients had a blood pressure increase of more than 50mmHg. Mean variation of heart rate in upright position was +12 beats per minute (standard deviation 20) but no functional symptoms were noted after standing up. Altered blood pressure circadian rhythm was also observed, the whole results suggesting an alteration of the autonomic nervous regulation of blood pressure [14]. The hormonal response to exercise was studied by Guenthard *et al*. in a group of 36 patients after coarctation repair. They observed a higher increase of plasma adrenaline after exercise in hypertensive patients. Thus, enhanced sympathetic nerve activity was proposed as a factor contributing to late hypertension after coarctation repair [15]. However, since no report of POTS following coarctation repair has been published yet to our knowledge, it is unclear if the results of these studies can be applied to our patient. Whether a state of augmented sympathetic activity and autonomic dysfunction related to aortic coarctation surgery could contribute to the development of POTS should be further evaluated.

UARS is a form of sleep-related breathing disorder belonging to the obstructive sleep-apnea and hypopnea syndromes (OSAHS). It is characterized by increased airflow resistance within the upper airways that leads to repetitive abnormal respiratory effort-related arousal. The consecutive sleep fragmentation gives rise to a range of symptoms similar to the usual form of OSAHS. UARS has been associated with symptoms common to diverse functional somatic syndromes, such as sleep-onset insomnia, headache, irritable bowel syndrome and depression [4]. In our patient, the presence of a micrognathia could partly explain the sleep disorder, but he never followed the recommendation to evaluate the necessity of using a mandibular propulsion device.

Is POTS more frequent in patients suffering from sleep-related breathing disorder? Guilleminault *et al*. [16] found a higher prevalence of low blood pressure and orthostatic intolerance among patients with UARS than among patients with the usual OSAHS. Interestingly, these authors studied 15 patients with UARS, low blood pressure and orthostatic intolerance with tilt-table testing and did not observe an abnormal heart rate after the maneuver in any of them. In a subsequent paper [17], they found signs suggestive of autonomic nervous system imbalance among the patients with a predominant parasympathetic tone. Our patient appears to have a very different behavior as he did not display any hypotension but presented abnormal tachycardia, which is consistent with predominant sympathetic rather than parasympathetic tone.

Are POTS patients prone to sleep-related breathing disorders? The association between UARS and POTS has never been explicitly reported, possibly because both entities have been described relatively recently. However, Bagai *et al*. found a higher prevalence of poor sleep quality in patients with POTS than in healthy control participants [2]. Additionally, there may be a substantial overlap between POTS, UARS and chronic fatigue syndrome. Orthostatic intolerance is frequent in patients with chronic fatigue syndrome and is related to autonomic dysfunction. In a recent study, POTS was a frequent finding in a cohort of patients with the clinical diagnosis of chronic fatigue syndrome [18]. Guilleminault *et al*. found a significant increase in the respiratory disturbance index in patients with chronic fatigue compared with controls, with the presence of increased respiratory effort and of flow limitation events in these patients [19]. This suggests that patients with chronic fatigue and UARS could share similar sleep disturbances. The relationship between UARS, POTS and chronic fatigue syndrome seems complex and incompletely understood. Further studies with objective measures like polysomnography are necessary to better characterize sleep disorders associated with POTS and the overlap that may exist between these various conditions.

## Conclusion

This report is the first, to the best of our knowledge, to document the occurrence of POTS after the surgical correction of an aortic coarctation. We also suggest that POTS and UARS can coexist in a patient. Based on the available literature, pathophysiological explanations may be attempted to characterize the nature of these associations, but whether causal relationships exist has still to be evaluated in future studies.

## Consent

Written informed consent was obtained from the patient for publication of this case report. A copy of the written consent is available for review by the Editor-in-Chief of this journal.

## Competing interests

The authors declare that they have no competing interests.

## Authors’ contributions

LF and MRN have drafted and revised the manuscript for important intellectual content. AC, SK, TR, SP and HB drafted specific parts of the manuscript and revised it critically. All authors read and approved the final manuscript.
